# Advancing the study of microbial symbionts of amphibians and reptiles

**DOI:** 10.3389/famrs.2026.1810202

**Published:** 2026-04-19

**Authors:** Margaret L. Doolin, Douglas C. Woodhams

**Affiliations:** Department of Biology, University of Massachusetts Boston, Boston, MA, United States

**Keywords:** amphibian, beneficial microbes, disease, microbiome, multi-omics, mycobiome, reptile

## Abstract

Amphibian and reptile microbiomes are important contributors to host health and ecosystem dynamics but understudied compared to microbiomes of other vertebrates. Through study of amphibian and reptile microbiomes, investigators can advance theory and practice in animal health, ecosystem function, conservation, and more. This special issue of *Frontiers of Amphibian and Reptile Science* features six publications in the field of amphibian and reptile microbiome research, and here, we highlight the contributions of these and other recent publications to four main areas of recent advancement in the field: 1) expanding “microbiome” beyond bacteria, 2) expanding study of host life-history stages and anatomical microbial habitats, 3) characterizing whole microbial communities, particularly in disease ecology studies, and 4) developing and implementing databases and bioinformatics tools. We also discuss several avenues for future research that would bring amphibians and reptiles to the forefront of microbiome research. With the continued implementation of classic culture-based and sequencing approaches paired with cutting-edge tools *in vivo, in vitro*, and *in silico*, herpetofauna microbiome research is poised for exciting growth in the near future.

## 1 Introduction

**FIGURE 1:**
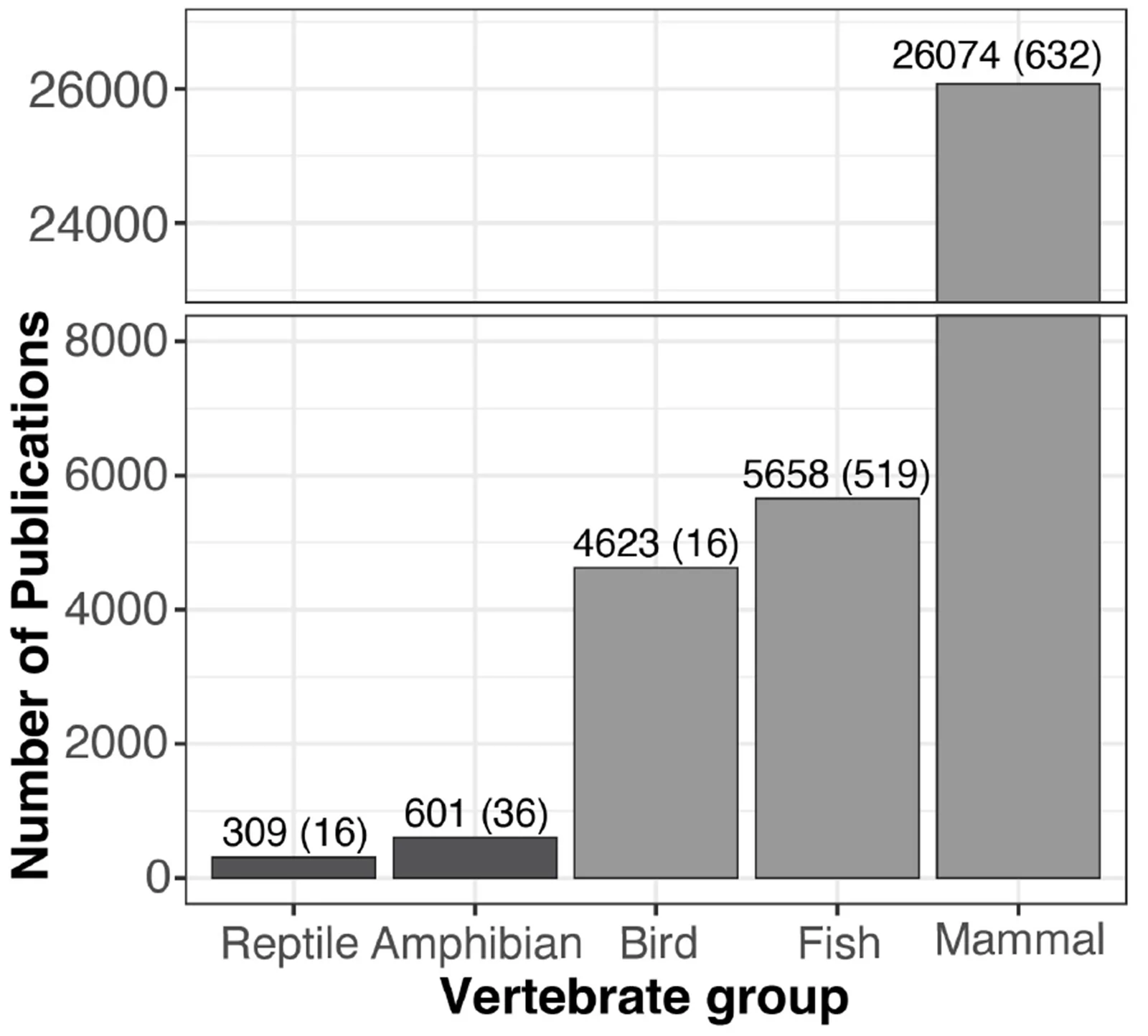
Quantification of “< vertebrate group> AND <microbiome>” searches in PubMed database. Numbers on top of each bar denote the total number of publications pertaining to the search including reviews, with the number of reviews in parentheses. Note that the mammal search excluded research on humans, and the amphibian search included *Xenopus*, axolotl, and *Ambystoma* to ensure that model organism research was captured. Search was completed on 27 Mar 2026.

The microbiomes of amphibians and reptiles are understudied compared to microbiomes of other vertebrates (Figure 1). These groups have been relatively overlooked due to a perceived lack of importance to anthropocentric interests, but amphibians and reptiles warrant dedicated study because they support ecosystem function and have physiologies that result in unique interactions with microbes (Gardner et al., 2007; Saha et al., 2018). Indeed, some species are already model systems for study of immunology and fungal pathogens (Field et al., 2022; Ruiz and Robert, 2023; Rollins-Smith, 2024). Most notably, amphibians face severe risks of past and future decline from disease caused by pathogenic *Batrachochytrium* fungi (chytridiomycosis; Luedtke et al., 2023), and reptiles face the emergence of snake fungal disease (ophidiomycosis; Lorch et al., 2016) and risks of opportunistic microbial overgrowth like mouth rot disease (infectious stomatitis) that are not faced by endotherms (Seyedmousavi et al., 2018). The benefits of studying herpetofauna-associated microbes include uncovering novel benefits of microbes (e.g., Jiménez and Sommer, 2017; Siddiqui et al., 2025), mechanisms of pathogen transmission (e.g., Ufermann et al., 2025), and methods to support animal health and conservation (e.g., Kueneman et al., 2016; Xiong et al., 2022). Considering the relative lack of study of amphibian and reptile microbiomes compared to other vertebrate groups, there are many topics that are unexplored or have only just begun to be addressed.

In recent investigations, including those in this special issue of Frontiers of Amphibian and Reptile Science, researchers have focused on diverse topics that are integral to advancing understanding of the roles that microbes play for amphibians and reptiles. Here, we highlight four topics that we deem to be important areas of recent growth in herpetofauna microbiome research. First, as called for by previous reviews (Walke and Belden, 2016; Jiménez and Sommer, 2017), more researchers have incorporated characterizations of non-bacterial microbial symbionts in addition to bacteria when characterizing the microbiome. Second, there has been more effort to study microbes across life stages and body sites to elucidate the complexity and specificity of amphibian and reptile microbiomes through time and space. Third, more studies have non-specifically characterized microbes to yield datasets including beneficial, pathogenic, and commensal microbes rather than a narrow focus on agents of disease. Finally, improved databases and methods related to microbial taxonomic and functional identification as well as bioinformatics tools have enabled more effective multi-dimensional and multi-omic studies. These advances have resulted in improved understanding of microbiome contributions to host and environmental health and a reduction in microbial dark matter associated with amphibians and reptiles.

## 2 Areas of recent and continuing advances

### 2.1 Expanding “microbiome” beyond bacteria

Studies of amphibian and reptile microbiomes focus primarily on bacteria, with recent effort to incorporate fungi (Kearns et al., 2017; Barnhart-McCarty et al., 2024; Vargas-Gastélum et al., 2024), microeukaryotes (Kueneman et al., 2017; Anslan et al., 2021; Bouchali et al., 2025), archaea (McGrath-Blaser et al., 2021; Madison et al., 2023), viruses (Harding et al., 2022; Li et al., 2024), or phage (Bueren, 2024; James et al., 2026). This bias is somewhat due to practical limitations of cost and efficiency associated with DNA sequencing because bacteria are typically the most abundant members of the microbiome and have, therefore, been regarded as the most important to characterize. DNA metabarcoding is most often used to characterize complex microbial communities, and the *16S rRNA* gene is an effective metabarcoding target to capture bacterial diversity that does not characterize most other microbes with reliable taxonomy identifications in existing databases. As sequencing costs continue to decrease, more studies may use multiple metabarcoding targets to capture more kingdoms of life, such as Barnhart-McCarty et al. (2024) have done in this special issue. The group amplified the internal transcribed spacer (ITS) region for fungi to describe a rich fungal community (“mycobiome”), in addition to amplifying the 16S rRNA gene for bacteria. Although Barnhart-McCarty et al. did not find significant associations with the fungal community and treatments in their study, other studies in amphibians (Kearns et al., 2017; Vargas-Gastélum et al., 2024) and other vertebrates (Bendová et al., 2020; Ost and Round, 2023) have found that fungal communities are putative supporters of immune function and digestion. It seems likely, therefore, that broader representation across kingdoms within amphibian and reptile “microbiomes” by using multiple sequencing targets would yield important findings related to host health and, especially, microbial diversity.

Multi-kingdom microbial diversity can also be assessed with methods outside of metabarcoding, with different benefits and detractors. For example, shotgun metagenomics garners information on the functional scope of the community in addition to taxonomy, which can be used to link microbial communities to animal health (*see* Bates et al., 2022 for amphibian example). Shotgun metagenomics overcomes shortcomings of DNA metabarcoding including amplification of off-target products (Klure et al., 2022) or missing important community members due to primer sequence mismatches (Op De Beeck et al., 2014; Stapleton et al., 2022) but is still subject to challenges of amplification biases, reliance on databases that may be data deficient (e.g., microeukaryotes), and relatively high cost per sample. Higher costs can pose an obstacle for groups studying amphibians and reptiles that do not have access to large funding sources like those applicable to human or translational medicine research. To this end, there is an emphasis on culture-based approaches to capture diversity in studies of amphibian and reptile microbiomes, in which bacteria and fungi can be cultured and assayed at low cost with high-impact advances on understanding microbiome composition and function (Brucker et al., 2008; Hill et al., 2018; Woodhams et al., 2020b; Sumanasekera et al., 2026). In this issue, Boulet et al. (2025) cultured bacteria and fungi from lizard egg clutches and carried out *in vitro* bacterial-fungal competition assays for a targeted approach to multi-kingdom characterization of host-associated microbiomes. By culture-based methods, this group found that soil fungi at lizard nesting sites are previously unrecognized infectious agents and interact with bacteria in maternal secretions. There is no single “best” approach to community characterization, but the movement toward broadening the investigations of the “microbiome” past bacteria will continue to yield a more complete understanding of community dynamics in microbes associated with amphibians and reptiles.

### 2.2 Expanding study of host life-history stages and anatomical microbial habitats

At different life stages, amphibians and reptiles face shifting immunological conditions that result in microbiome shifts. At the egg and hatchling stages, all oviparous animals are vulnerable to microbial overgrowth due to limited immune protection and mobility, but there is evidence that maternal microbes (Walke et al., 2011) and egg laying behavior (Banning et al., 2008) can boost protection against pathogens, in addition to endogenous immune or developmental processes (Gomez-Mestre et al., 2006; Brown and Shine, 2022). In this issue, Boulet et al. (2025) highlight that even reptiles with no parental care can protect eggs from fungal overgrowth via bacteria in maternal cloacal secretions. Furthermore, the vulnerability to microbial establishment in early life is not always negative, as highlighted in this issue by Wallace et al. (2025) showing the specificity with which the symbiotic alga *Oophila* colonizes spotted salamander embryos for a metabolic benefit. Unique among vertebrates, amphibians also have a second opportunity for large microbial shifts and colonization at metamorphosis. At this time of physical and physiological change, the immune system undergoes modifications, and there is depressed immune function that is the likely cause of poor colonization resistance to microbes (Humphries et al., 2022, Humphries et al., 2025). The vulnerability to microbial colonization at metamorphosis as well as embryonic or hatchling stages could be harnessed to promote animal health by applying exogenous probiotics to combat disease, as has been attempted with mixed success (Jones et al., 2024).

Certain anatomical regions or microhabitats on amphibians and reptiles are also special areas of research focus due to being sources of unique microbes or sites of important microbial interactions (Ghose and Eisen, 2025). In reptiles, studies have elucidated powerful functional links between the gut microbiome and host characteristics (e.g., associations with personality [Harlow et al., 2025], herbivorous digestion [Vasco et al., 2022]), and sourced unique microbes from sites of special interest in charismatic species (e.g., salivary microbiome of Komodo dragons [Hyde et al., 2016], oral microbiome of venomous snakes [Chuang et al., 2022]). In contrast, amphibians are unique among vertebrates in that the majority of microbiome studies on amphibians has characterized the skin microbiome due to the importance of the skin in amphibian health [e.g., 315/601 publications compared to 25/309 in reptiles based on PubMed search of (<group> AND microbiome AND skin)/(<group> AND microbiome)]. This bias is exemplified by the six publications in this special issue, in which three publications focus on amphibian skin microbiomes (Barnhart-McCarty et al., 2024; Woodhams et al., 2024; Jost et al., 2025), one on amphibian skin and cloacal microbiomes (Scarberry et al., 2024), one on amphibian subcutaneous microbes (Wallace et al., 2025), and one on lizard cloacal microbes (Boulet et al., 2025). Studies of the amphibian skin microbiome continue to contribute important insights due to its applicability to animal health and lack of study of skin microbes in other groups. Nonetheless, research into understudied tissues that are vulnerable to microbial colonization (e.g. tadpole brains; Emerson et al., 2025) or manipulation of host-associated microbes throughout life history stages (Knutie et al., 2017; Warne et al., 2019; Miller et al., 2023) may yield further exciting insights into unique microbiome dynamics across body sites.

### 2.3 Characterizing whole microbial communities

The study of microbes associated with amphibians and reptiles has long been driven by studying agents of disease, but there is much to discover about microbial community dynamics and the roles of non-pathogenic microbial symbionts in disease and other conditions (Méthot and Alizon, 2014; Wiles and Guillemin, 2019). Since calls began for a more complete understanding of the diversity and function of residential amphibian and reptile microbiomes (e.g., Colston and Jackson, 2016), many groups have characterized whole bacterial communities for broad perspectives on microbial community dynamics. In this special issue, four out of six publications present untargeted characterizations of bacterial communities through DNA metabarcoding to yield insights into host-bacterial interactions (Woodhams et al., 2024; Jost et al., 2025), fungal-bacterial interactions (Barnhart-McCarty et al., 2024; Woodhams et al., 2024), and conservation implications of microbial community composition (Scarberry et al., 2024). In other recent research that incorporates host characteristics, the link between whiptail lizard genetics and microbiome composition has the potential to revolutionize understanding of microbiome assembly and host-microbe interactions (Camper and Bewick, 2025; Camper et al., 2025). Indeed, amphibian and reptile microbiome research is ripe for contributions to several cutting-edge areas of microbiome research with broad characterization of microbiome and host characteristics. Some researchers have started investigations into topics such as early-life disruption of microbiota and developmental windows impacting disease susceptibility (Knutie et al., 2017; Warne et al., 2019; Miller et al., 2023), microbiome-mediated heat tolerance in light of climate change (Fontaine and Kohl, 2023), and the adaptive microbiome hypothesis and ecological resilience (Woodhams et al., 2023) that hold promise for exciting contributions in the future. With approaches that combine microbiome, host, and environmental inputs, researchers will better tease apart the dynamics of herpetofauna microbiomes with findings informative across vertebrates.

### 2.4 Developing and implementing databases and bioinformatics tools

Although amphibian and reptile microbiomes have been studied less than other vertebrate groups, researchers capitalize on databases and approaches applicable across systems for successful multi-omic studies. In this special issue, Jost et al. (2025) provide an excellent example of researchers using established methods from disparate fields to generate a multi-omic dataset on new species. Their work relied on advanced chemical analysis, *in vitro* fungal culture, microbiota characterization with the QIIME2 bioinformatic pipeline (Bolyen et al., 2019), and taxonomic identifications with SILVA (Quast et al., 2013). Also in this issue, Barnhart-McCarty et al. (2024) used the UNITE database (Abarenkov et al., 2010) for identification of *ITS* amplicon sequences of fungal communities, and Boulet et al. (2025) annotated bacterial genomes from lizard eggs using UniProt to identify putative proteins and antiSMASH (Blin et al., 2019) to identify genes with links to synthesis of antimicrobials. None of these tools or databases was created for amphibians or reptiles, but all serve as extremely important and reliable tools in herpetofauna microbiome research.

A broad range of microbiome analysis packages and approaches are incorporated into studies of amphibian and reptile microbiome research. Trait-based analyses on cultures can be very useful (e.g., BacDive [Schober et al., 2025], Omnicrobe [Dérozier et al., 2023]). Other tools for analyzing microbiomes include the 4H-index developed for examining hybrid lizard microbiomes (Camper et al., 2024), co-occurrence of microbes (Griffith et al., 2016; Rivett and Bell, 2018; Liu et al., 2023), microbial centrality analyses (Rawstern et al., 2025), core microbiomes (*see* Risely, 2020), or combinations of tools for measuring microbial diversity-stability dynamics with infection (Harrison et al., 2019) and ecological modeling (e.g., *glmmTMB* [McGillycuddy et al., 2025]), Joint Species Distribution Models [Ovaskainen et al., 2017], Dynamic Covariance Mapping [Gencel et al., 2025]). This is a rapidly developing bioinformatics field with machine learning and additional artificial intelligence approaches on the horizon (Rizzi et al., 2025) that will continue to fill gaps in existing tools.

**FIGURE 2:**
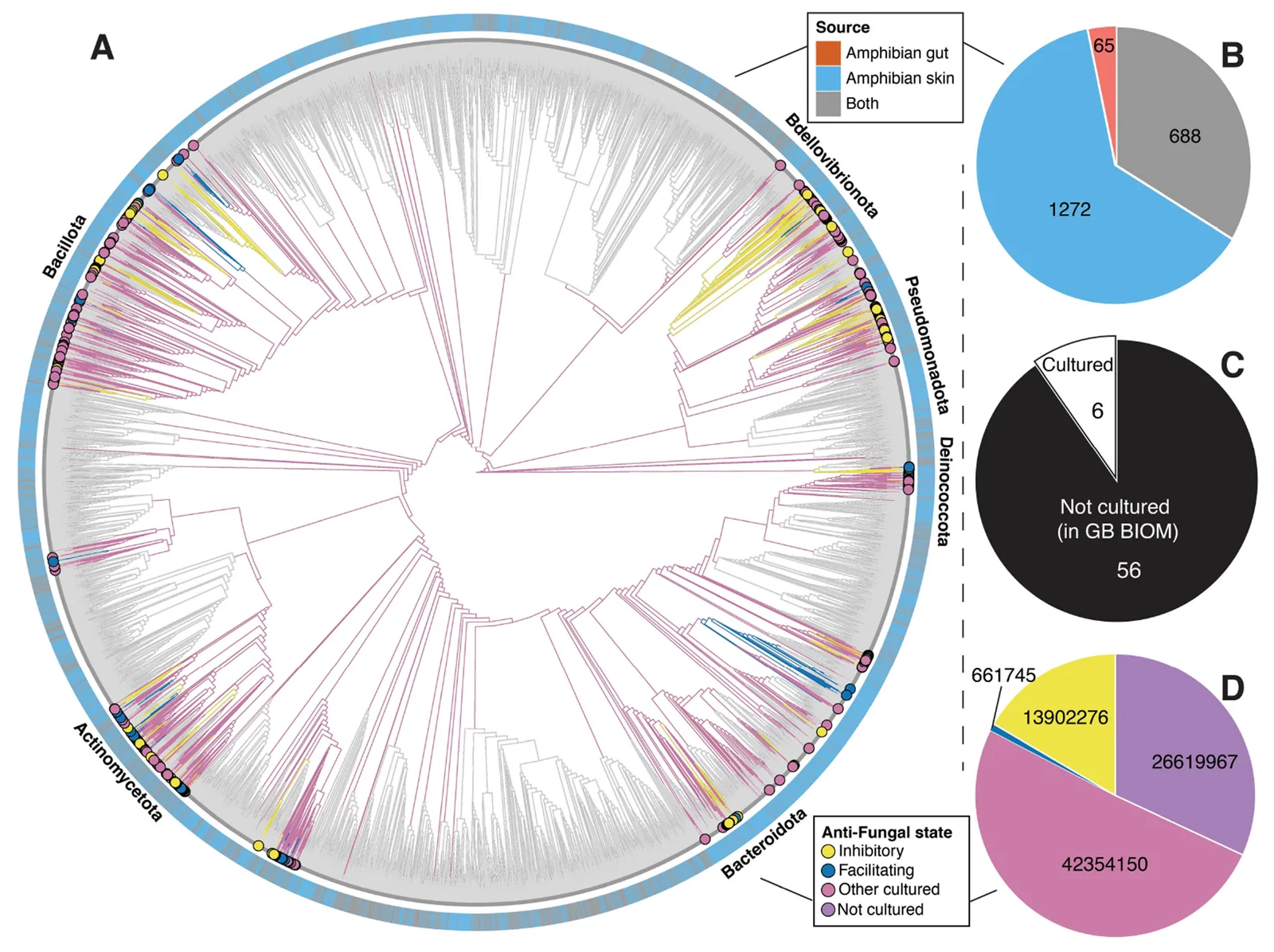
Application of amphibian-associated bacterial culture database (AmphiBac 2025.1) to studies of amphibian-associated microbiomes. Approximately 92% (8024/8739 isolates) of AmphiBac amplicon sequence variants (“ASVs,” *i.e.*, unique *16S rRNA* gene sequences) matched to a meta-analysis of 3601 amphibian skin microbiome samples and 219 amphibian gut microbiome samples from 211 amphibian species, at a 97% similarity threshold. **(A)** Phylogenetic tree, where colors on branches and nodes denote anti-fungal state and colors on outer ring heatmap denote sample source. **(B)** The proportions of bacterial genera represented in gut samples, skin samples, or both, labeled with number of genera in each slice. **(C)** The proportions of bacterial phyla with representatives in culture or not in culture, noting that 56/62 phyla represented have not been cultured. **(D)** The relative abundance of amphibian skin-associated bacterial reads assigned as inhibiting, facilitating, not interacting with, or not cultured (i.e., unknown function) regarding the growth of *Batrachochytrium* fungi. All data were previously published and aggregated by Woodhams et al. (2020a), and this application of the AmphiBac database was completed for this publication.

It is also worth noting that some group-specific tools for microbiome research have not included applicable data for amphibians and reptiles, and creating new tools and datasets that specifically target herpetofauna have enabled important advances. For example, Woodhams et al. (2024) benefited from a wealth of past investigations into host defense peptides secreted by amphibians so that they could identify peptides from a new population of frogs in Panama. The AmphiBac database is another impactful tool of broad interest across amphibian microbiome research (Woodhams et al., 2015; Bletz et al., 2025), through which thousands of bacteria are identifiable by their *16S rRNA* gene sequence and catalogued as inhibitory or non-inhibitory of pathogenic *Batrachochytrium* fungi based on *in vitro* culture assays. Users can categorize their own samples as likely or unlikely to inhibit *Batrachochytrium*, as has been done in this issue (Barnhart-McCarty et al., 2024; Woodhams et al., 2024) and elsewhere (*e.g.*, Siomko et al., 2023). As part of this review, we applied AmphiBac categorizations to a meta-analysis of host-associated microbiomes (Woodhams et al., 2020a) and found that most abundant taxa from amphibian skin were represented in the AmphiBac database, but that there are considerable gaps in predictive power for anti-*Batrachochytrium* function of rare taxa due to bacteria that have not yet been cultured (Figure 2). Continued contributions of reptile and amphibian samples to a breadth of databases, alongside machine learning, will improve reference points for future work.

## 3 Discussion

Recent advances in amphibian and reptile microbiome research have been broad across host species, research methods, and research goals. In this review, we focused on areas of research advancement related to the six articles in this special issue to situate the contributions of these publications among other research. As microbiomes of amphibians and reptiles remain the least studied of vertebrate microbiomes, fruitful discoveries undoubtedly await researchers who continue to contribute to the advancement of the field in both well-established and innovative ways. Future studies will improve understanding of the taxa specific to that work and build a larger body of work to more generally bridge gaps in understanding of understudied host-associated microbes. There are a handful of areas for research expansion in reptile and amphibian microbiomes that we believe could be especially useful to continue advancing this understanding.

One area of research that is greatly needed is focused study of overlooked microbial associates, especially the interactions among different groups of microbes on a host. How are bacteriophages influencing the health and ecology of their eukaryotic hosts (Bueren, 2024)? What role do algae play across developmental stages (Anslan et al., 2021; Wallace et al., 2025)? What kinds of viruses, archaea, and predatory microbes are active in amphibian and reptile microbiomes? For example, we recently discovered the amoeba *Capsaspora* spp. on the skin of adult bullfrogs, *Rana catesbeiana*, in Pennsylvania (P.J. Kearns, unpublished), which may be a predator of parasitic helminths like its congener *Capsaspora owczarzaki* (Kidner et al., 2024). Also, reptiles are recognized to host diverse microeukaryotes in their guts that have received little attention (San-Martín-Órdenes et al., 2019; Gerrick et al., 2023). Cross-referencing virological and parasitological literature could be avenues to recognizing multi-kingdom diversity in the microbiome, along the spectrum of symbiosis.

The functional landscape of microbes is also often overlooked in host-associated microbiome research. We encourage researchers to include direct measurements of microbiome function, especially at the community level, to improve understanding of microbe-microbe and host-microbe functional networks. Tools like PICRUSt2 (Douglas et al., 2020) or Piphillin (Iwai et al., 2016) are useful for predicting bacterial functions by proxy, but having a direct functional metric of microbes associated with a host can advance understanding past the available databases and generate unquestionable functional data that can be mined for future work. These measurements could include implementations of transcriptomics, proteomics, or metabolomics, and can be coupled with quantitative abundance measurements of microbes for the most impact (Wang et al., 2021). Investigators may consider *in vitro* approaches to acquire data in these types of -omics approaches due to abundance of host-derived signal *in vivo* and to reduce complexity of the study system.

Reducing complexity of systems by microbial manipulations in the lab is also a useful approach in whole-animal experiments. One area that is almost unexplored in amphibians and reptiles is generation of gnotobiotic and germ-free animals. Although this is a reductionist approach that changes the host system to some extent (Høverstad and Midtvedt, 1986; Tlaskalová-Hogenová et al., 2011), insights into host health and various types of treatments (*e.g.*, probiotics, pharmaceuticals, behavioral, etc.) gained from experimental work with gnotobiotics and germ-free animals compared to wild type animals are invaluable. Generation of germ-free amphibians is especially challenging due to an apparent reliance on microbes for development (*see* Miller et al., 2023), but generating reproducible germ-reduced animals is an achievable goal that will result in a wealth of knowledge, especially for immunology, metabolism, and development.

Finally, investigations in areas of microbiome research in which amphibians and reptiles are uniquely positioned to answer questions would further emphasize these groups as unique and valuable to the research community. For example, amphibian skin is an ideal model to study microbial roles in mucosal immunology (*e.g.*, Madison et al., 2025; Muletz-Wolz et al., 2025) and production and tolerance of toxins like tetrodotoxin (Jost et al., 2025), and lizards that readily hybridize provide a unique host background for understanding microbiome assembly (Camper et al., 2025). To reduce reliance on conservation-sensitive host species, some studies could be carried out *in vitro* using synthetic communities (*i.e.*, model microbial communities assembled from individual cultures) or host cell cultures (Bui-Marinos et al., 2022). Other needed improvements in tools for amphibian and reptile microbiome research include targeting of bacteria that have yet to be cultured with helper strains, use of selective media (Koblitz et al., 2023), or novel single cell (Lloréns-Rico et al., 2022) and high-throughput culturomics approaches (Huang et al., 2023).

There are many promising research directions for investigations of microbiomes of amphibians and reptiles. Whether addressing our suggested outstanding topics for research advancement or contributing additional research using traditional methods, additional investigations of herpetofauna microbiomes will continue to propel microbiome research forward with exciting findings relevant to animal health, ecosystem function, conservation, and more.
